# Ageism in the Discourse and Practice of Designing Digital Technology for Older Persons: A Scoping Review

**DOI:** 10.1093/geront/gnac144

**Published:** 2022-09-21

**Authors:** Ittay Mannheim, Eveline J M Wouters, Hanna Köttl, Leonieke C van Boekel, Rens Brankaert, Yvonne van Zaalen

**Affiliations:** School of Allied Health Professions, Fontys University of Applied Science, Eindhoven, the Netherlands; Tranzo, School of Social and Behavioral Sciences, Tilburg University, Tilburg, the Netherlands; School of Allied Health Professions, Fontys University of Applied Science, Eindhoven, the Netherlands; Tranzo, School of Social and Behavioral Sciences, Tilburg University, Tilburg, the Netherlands; Louis and Gabi Weisfeld School of Social Work, Faculty of Social Sciences, Bar-Ilan University, Ramat Gan, Israel; Department of Health Sciences, IMC University of Applied Sciences Krems, Krems an der Donau, Austria; Tranzo, School of Social and Behavioral Sciences, Tilburg University, Tilburg, the Netherlands; School of Allied Health Professions, Fontys University of Applied Science, Eindhoven, the Netherlands; Expertise Centre Dementia and Technology, University of Technology Eindhoven, Eindhoven, the Netherlands; School of Allied Health Professions, Fontys University of Applied Science, Eindhoven, the Netherlands

**Keywords:** Ageism, Critical discourse analysis, Digital technology, Participatory design, User-centered design

## Abstract

**Background and Objectives:**

Involving older persons in the design process of digital technology (DT) promotes the development of technologies that are appealing, beneficial, and used. However, negative discourse on aging and ageism are potential underlying factors that could influence which and how DTs are designed and how older persons are involved in the design process. This scoping review investigates the explicit and implicit manifestations of ageism in the design process of DT.

**Research Design and Methods:**

Seven databases were screened for studies reporting on the design of DT with older persons between January 2015 and January 2020. Data regarding study and DT characteristics, discourse about older persons, and their involvement in the design process were extracted, coded, and analyzed using critical discourse analysis.

**Results:**

Sixty articles met the inclusion criteria and were included in the analysis. Various forms of exclusion of older persons from the design process were identified, such as no or low involvement, upper-age limits, and sample biases toward relatively “active,” healthy and “tech-savvy” older persons. Critical discourse analysis revealed the use of outdated language, stereotypical categorizations, and/or design decisions based on ageism in 71.7% of the studies.

**Discussion and Implications:**

A discrepancy was found between an “ideal” discourse regarding the involvement of older persons throughout the design process and actual practice. Manifestations of ageism, errors, and biases of designing DT with older persons are discussed. This article calls for more authentic inclusion of older persons and higher awareness toward the implications of ageism in the design process of DT.

The vast development of digital technology (DT) offers potential benefits in various life domains, such as socialization, leisure, working environments, commerce, and health care. The opportunities of developing DT to address the needs of older persons and improve their well-being are often emphasized in research and policy (Schulz et al., 2015), and it is widely recognized that in order to design relevant DTs, that are eventually used, end-users should be involved throughout the design process (Fischer et al., 2020; Gómez & Criado, 2021; Sanders & Stappers, 2008). Simultaneously, chronological age is often discoursed and mentioned as a barrier of technology adoption (Venkatesh et al., 2012), and ageism might indeed persist as a factor impinging on the design of DT and actual use (Lee & Coughlin, 2015; McDonough, 2016). This, in turn, could influence what types of DTs are designed, the needs they meet and how they are used (Peine & Neven, 2021). However, little is known about how ageism might manifest in the design process of DT.

Ageism comprises stereotypes, prejudice, and discrimination toward a person based on their age (Officer & de la Fuente-Núñez, 2018). Swift et al. (2017) found that three mechanisms of ageism can negatively influence active aging and social participation. Namely, stereotype embodiment (the process of internalizing age stereotypes and applying them to oneself); stereotype threat (avoiding a stereotypical domain from the fear of conforming to negative age-stereotypes); and age discrimination. Empirical evidence of ageism in the context of using DT is still limited (Köttl et al., 2022). Nevertheless, recent findings suggest that experienced ageism, as well as self-ageism and stereotype threat, may lead to lower use of DT and a widening of the digital divide (Caspi et al., 2019; Choi et al., 2020; Köttl et al., 2021; Mannheim et al., 2021; Mariano et al., 2020; Rosales & Fernández-Ardèvol, 2020; Xi et al., 2021, 2022). Furthermore, ageism may influence how older persons’ motivations and abilities to use DT are perceived by others (Lee & Coughlin, 2015; McDonough, 2016).

Ageism in the design process manifests in different forms. Importantly, ageism can operate in an implicit and unaware manner (Levy & Banaji, 2002), and without the intention to do harm (Swift et al., 2017). Thus, designers and researchers with positive intentions to improve the well-being of older persons, might not be aware of how ageism biases the design process of DT. To begin with, the perspectives of designers and other stakeholders are different from those of older persons, intended to use the DT (van Boekel et al., 2019). A common discourse regarding DT for older adults, is mainly referring to the negative aspects of aging, and associating aging mainly with frailty, cognitive decline, and dependency. Indeed, avoiding categorizations of older adults and acknowledging the diversity and multifacet identity of older persons, seems to be a potential pitfall for many designers in the design process (Righi et al., 2017). In a critical discourse analysis of 30 years of publications about aging in Human–Computer Interaction, Vines et al. (2015) identified that aging was mainly framed as a problem, and discoursed predominantly on societal and economic consequences of health and care needs of older persons. Consequently, DT is often seen as an intervention for managing the challenges of aging (Peine & Neven, 2019).

Categorizing and imagining older adults as needing help and having deteriorating health may lead to a fixation on health care-related DT (Schulz et al., 2015). Yet, this is often in contrast to older adults’ life realities. Evidence shows that many older adults do not perceive themselves as unhealthy, hence declining the need for assistive and health-related DT (Claes et al., 2015). Furthermore, older adults have other motivations and desires for DTs that meet social, leisure, and hedonic needs (Astell, 2013). In their model of coconstitution of aging and technology (CAT), Peine and Neven (2021) elaborate on the interactive cycle in which “images of aging” influence the “design worlds” in the decision-making process of designing DT. Assumptions of older persons as users are embedded in the designed “technological artifacts,” which in turn influence how older persons use them. Certain assumptions, drawn-out without the input of older persons, may lead to designing DTs that are stigmatizing (Köttl et al., 2021), aesthetically unappealing (de Jonge et al., 2016), “script” use-characteristics such as giving up autonomy to others (Peine & Neven, 2021), and eventually lead to low adoption rates and abandonment of DTs (Greenhalgh et al., 2017).

The principle of involving end-users throughout the design process of DT is emphasized by many studies (Brankaert & den Ouden, 2017; Fischer et al., 2020; Sanders & Stappers, 2008; Steen et al., 2011) as well as industry (ISO, 2010). Theory and methods of designing and involving end-users have developed over the past years. Main concepts include user-centered design theories (e.g., Norman, 1986), mainly deriving from North America, and participatory design (Muller & Kuhn, 1993), mainly developed in (Northern) Europe. These approaches have started to influence each other (Sanders & Stappers, 2008), and new developments are emerging, such as codesign (Steen et al., 2011) deriving from participatory design and emphasizing engaging participants in the design space. These approaches collectively emphasize the importance of involving the most relevant target group, in an iterative nature throughout various stages, in order to learn about the lives, wants, and needs of end-users. Doing so is thought to lead to designing DTs that are relevant for the end-users’ purposes and are actually used. Importantly, it is increasingly recognized that people are not just “end-users” or participants, who are mainly observed and researched (Corrado et al., 2020). Rather, they are experts of their own experience, capable of creativity (Sanders & Stappers, 2008), and have a central role in how DT is actually used (Peine & Neven, 2021).

Nevertheless, older persons are often excluded from the design process of DT (Mannheim et al., 2019), which may be considered a form of discrimination. Simply involving older adults in the design process does not guarantee that the design process is bias- and ageism-free, and that older persons have an actual say in designing the outcome. Typically, design processes include phases of empathizing, prototyping, testing, and evaluating the outcomes. In a previous literature review, Fischer et al. (2020) explored the perceived importance and motivation of involving older persons in technology design and the nature of their involvement. It was found that the majority of studies involved older persons in the initial and/or final stages of the design process, however, much less during design practices (e.g., prototyping). Only 10% of studies involved older persons in all stages of the design. As aforementioned, involving the end-users throughout the whole design process may increase the chance of designing DTs that are used and adopted. Accordingly, potential biases would be involving older adults only in parts of the design process, without iteration or continuation (Frennert & Östlund, 2014); selection biases in choosing participants who do not represent older end-users, for example, including mainly healthy active participants (Howes et al., 2017); or selectively implementing or ignoring the say and feedback of older persons (Fischer et al., 2020).

Fischer et al. (2020) addressed designers’ motivations and perceived benefits for older persons’ involvement. However, negative aspects influencing the design of DT, such as ageism are usually not self-reported because the concept of ageism might be a latent or implicit variable in the design process. Therefore, this current scoping review attempts to take a step forward by critically addressing: what are the explicit and implicit manifestations of ageism in the design process of DTs intended for the use of older persons? Additionally, this study will attempt to answer the following subquestions:

(1) What are the underlying assumptions about older persons and their abilities to contribute to the design process? How is this embedded in the discourse about aging?
(2) To what extent are older persons included in the design process of DT? Who are the older persons that are involved? And what is the nature of their involvement?
(3) If older persons are included in the design process, how does ageism shape the design process of DT?

## Method

### Design

Scoping reviews are commonly used to address gaps in the literature and assess the extent, range, and nature of the evidence on a topic or question (Arksey & O’Malley, 2005). This study aims for a thorough, reliable, and comprehensive approach. Therefore the checklist of the Preferred Reporting Items for Systematic reviews and Meta-Analyses extension for Scoping Reviews (PRISMA-ScR; Tricco et al., 2018) was followed.

### Search Strategy

As the topic of aging, design, and technology crosses different disciplines, our search took place in databases related to health care, psychology, sociology, and design of technology: PubMed, Ageline (EBSCO), CINAHL (EBSCO), ACM Digital Library, Web of Science, and PsycINFO. Due to the specific interest of this study in participatory- and codesign, the journal of Codesign (listed by Taylor & Francis) was also screened. The search was conducted during the 10th–17th of January 2020 and included three main concept terms connected by the “AND” Boolean: Design, Aging, and Digital Technology. Each concept comprised of related terms and synonyms (Online Supplementary Material Section 1) connected by the “OR” Boolean. Synonyms were developed through discussions and related literature and were iteratively tested on PubMed to maximize the results of the search string. The concept of ageism was not used as a separate concept, as studies usually do not self-report ageist attitudes. Indeed, initial search attempts revealed nearly zero results with this combination. Thus, the concept of Ageism was added with the “OR” Boolean to the concept of Aging. As the term DT can lead to a vast range of definitions, we added specific terms of DT in the context of aging (e.g., “robot” and “age-tech”). Generally, we applied the search of the terms for titles and abstracts. The search string was modified according to each database (Online Supplementary Material Section 2).

### Inclusion–Exclusion Criteria

We included peer-reviewed studies in English, reporting at least part of the design process of a DT intended for the use of older persons (including informal caregivers who are often older persons themselves). Studies about DTs for the use of others (e.g., formal caregivers or health care professionals) were not included. We defined DT as technological devices, services, or platforms that use, collect, and often process data and are connected to the internet, other devices, or apps. Papers clearly reporting only an evaluation of a fully designed DT or the effect of using DT in interventions were excluded. The term “older person” was not strictly defined by a chronological age limit. Hence, studies were included if the authors of the study defined the target population as old or the designed DT was intended for a medical condition related to an older age. Due to the vast development of DT, we included literature from January 2015 until January 2020. Furthermore, reviews, opinion papers, and nonpeer-reviewed conference proceedings were excluded, as well as studies that only reported fundamentally technical or theoretical aspects of the DT.

### Selection Process

The **s**election process was performed using “Covidence,” an online tool for conducting literature reviews. After screening for duplicates, all titles and abstracts were screened according to the inclusion criteria by two independent assessors. I. Mannheim screened all studies, while the second independent assessment was equally divided by all other coauthors (E. J. M. Wouters, H. Köttl, L. van Boekel, R. Brankaert, and Y. van Zaalen). As exclusion from the design process may be considered a form of discrimination, studies reporting a design process of DT intended for the use of older persons, which clearly did not involve them (e.g., involving only professionals), were excluded with the tag of “designed without older adults.” Following, full texts were screened by two independent assessors, and data were extracted. I. Mannheim screened and extracted all studies; the second screening assessment was equally divided between all other coauthors. Disagreements in both phases were discussed and, if needed, disputed by a third assessor.

### Data Extraction and Analysis

Data were extracted by two independent assessors and subsequently compared for consistency and richness of content. In order to address implicit and explicit manifestations of ageism in the design process, characteristics of the design process were extracted, such as the type of DT, the nature of how older adults were sampled and involved (sampling criteria, stage of the design process, number of design sessions, and iterative approach), incorporation of feedback and characteristics of the participants and the studies.

Importantly, as we were interested in identifying possible manifestations of ageism through the discourse about aging, we extracted segments of text regarding the discourse on assumptions of the researchers and designers about older persons as participants in the design process and end-users; discourse and conclusions on the adoption of technology by older persons; and descriptions on the nature of involvement of older persons in the design process. Direct mentioning of ageism was also extracted, such as the use of outdated language (e.g., “elderly” and “aging tsunami.” See an example of adopting new AMA guidelines in Lundebjerg et al., 2017), use of categorizations about older adults that could be stereotypical, as well as documentation of design decisions that seemed to be based on stereotypes.

Inspired by critical discourse analysis (CDA; Fairclough, 2003; Van Dijk, 1993), extracted data and text segment quotes were coded and analyzed. By applying CDA, we wanted to uncover the attitudes reflected in the choice of words and language. Viewing ageism as a currently socially accepted form of inequality, we sought to reveal the relations between the discourse on aging and DT and the power and dominance of designers and stakeholders in the design process. More specifically, in the coding process, we sought to identify attitudes, beliefs, and decisions in a way that could be somewhat quantified and comparable (e.g., how many studies mention the importance of involving older persons throughout the design process comparing to their actual involvement). Accordingly, we deepened into the discourse and choice of language (Online Supplementary Material Section 3 for the full list of extracted variables, quoted segments, and coding).

By choosing a critical approach, we clearly make a stance in uncovering social inequalities (Van Dijk, 1993) and what we believe to be harmful implications of ageism. Nevertheless, we also took measures to reflect on our own potential bias as researchers on ageism. In order to increase the credibility and trustworthiness, we held several calibration sessions with all authors regarding the selection process of the studies and to examine the analysis and interpretation of ageist discourse.

## Results

### Selection Process

**Figure 1.**
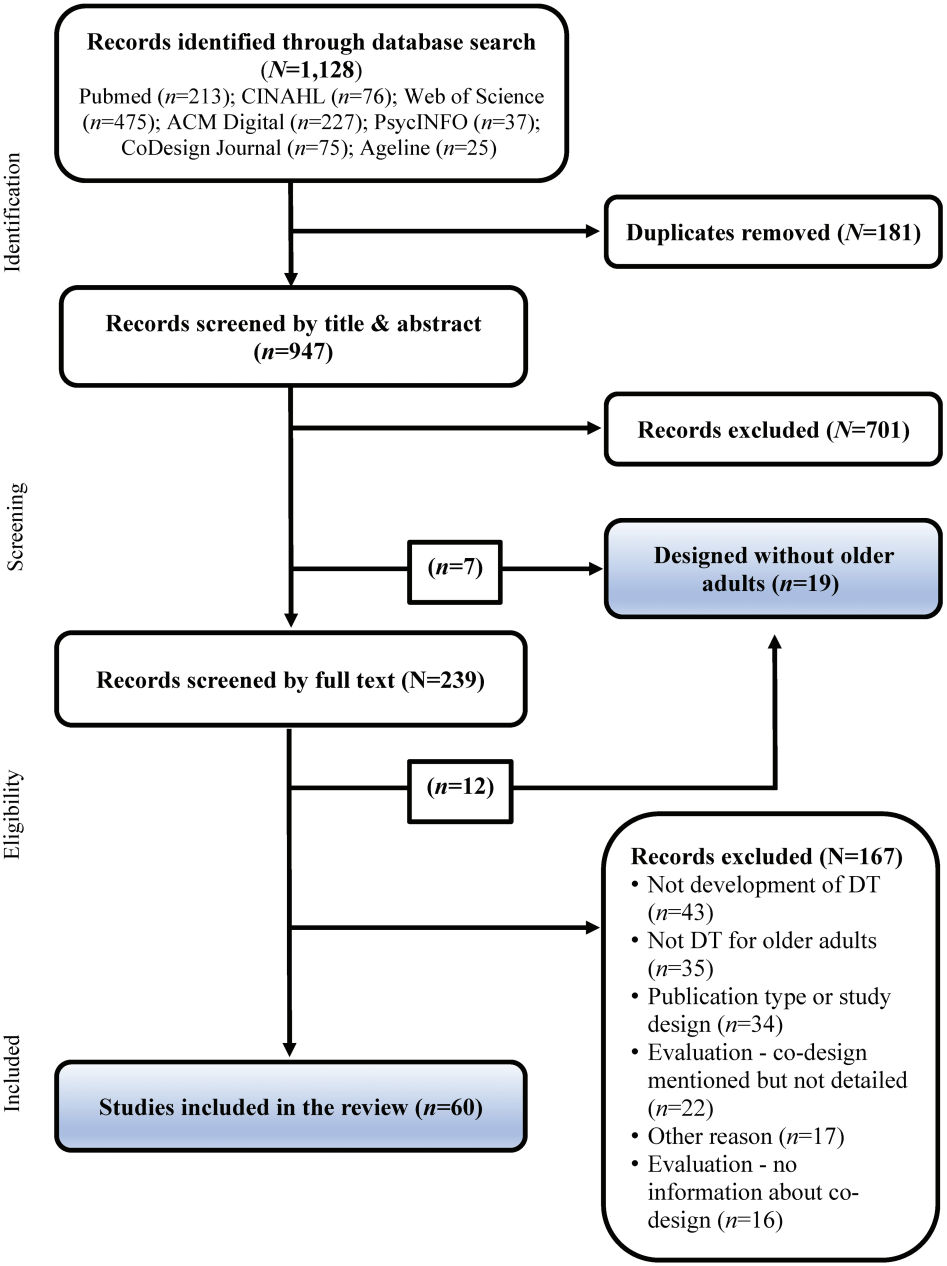
Flowchart of the selection process. Adapted and modified from (Tricco et al., 2018). Articles that excluded older persons from the design process all together were highlighted due to exclusion being a potential form of ageism in the design process. These articles were subsequently excluded from the review. DT = digital technology.

A total of 1,128 articles were identified (Figure 1). During the title and abstract phase, seven articles were identified in which DTs were clearly designed without older persons. Seven hundred and one additional papers were excluded for not meeting any of the other inclusion criteria. The remaining papers were subsequently screened for full-text eligibility. Twelve additional studies were identified as designing without older adults, leading to a total of 19 articles that excluded older persons entirely from the design process of DT and were thus highlighted in the selection process as “designed without older adults.” One hundred sixty-seven additional papers were excluded in the full-text eligibility phase. Of them, 38 studies (22.8%) were excluded because older persons were involved only in the evaluation of a fully designed DT and not in the design process. Of these studies, 22 indicated some sort of prior design process involving older persons but provided no evidence of it, and 16 studies did not indicate that older adults were involved in the design process; however, it could also not be clearly concluded that older persons were excluded. Finally, 60 studies were included in the review.

### Characteristics

A summary of the studies’ characteristics can be found in Table 1, and the full detail of extracted information can be found in Online Supplementary Material Section 3. Studies varied in providing adequate details regarding participants. In 55% of the studies, we defined the characteristics as adequate (e.g., providing at least details about the age and gender of all participants), in 23.3% characteristics were partially described, and in 21.7% the level of detail was found to be mostly missing (e.g., no basic details on age or gender). Participants involved in the design process varied from  2 to 1,346 per study. Only 33.4% of the studies included 10 or less participants and 60.1% of studies included 30 or less participants. Participants were predominantly female, with 53.4% of the studies having more than 50% female participants. Ten studies (16.7%) did not report the participants’ gender. Mean age of participants (indicated in 33 out of 60 studies) was 69.63. The mean age range (indicated in 44 studies) was 57.56–82.63. In eight studies (13.3%), no information about the age of participants was provided. In most studies (55%) participants were living independently at home. Only 33.4% did not clearly indicate living contexts.

**Table 1.** Study and Participants’ Characteristics

Variable	% (Number of studies)
Region (Country)	
Europe	66.7 (40/60)
North America (USA and Canada)	26.7 (16/60)
Asia (China, Malaysia, Taiwan, and Japan^a^)	6.7 (4/60)
Oceania (New Zealand)	1.7 (1/60)
Publication year	
2015	13.3 (8/60)
2016	11.7 (7/60)
2017	13.3 (8/60)
2018	28.3 (17/60)
2019	33.4 (20/60)
Ethical approval obtained	
Yes	60.0 (36/60)
Not indicated	35.0 (21/60)
Indicated as not required/other type of approval	5.0 (3/60)
Total participants^b^	
5 or fewer	6.7 (4/60)
6–10	26.7 (16/60)
11–30	26.7 (16/60)
31–100	25.0 (15/60)
More than 100	13.3 (8/60)
Not indicated	1.6 (1/60)
Living situation	
Community-dwelling	55.0 (33/60)
Nursing homes/assisted living	3.3 (2/60)
Community and nursing homes	8.3 (5/60)
Not indicated/not clear	33.4 (20/60)
Gender (% female)	
Less than 25%	8.3 (5/60)
25%–50%	21.6 (13/60)
51%–75%	36.7 (22/60)
More than 75%	16.7 (10/60)
Missing data	16.7 (10/60)
Age	
Mean (*N*_studies_ = 33; *N*_particpants_ = 1,897)	69.63
Mean Range (Min–Max; *N*_studies_ = 44)	57.56–82.63
Median (*N*_studies_ = 9)	71.15
Missing data on participants age	13.3 (8/60)
Education	
Mainly higher education	16.7 (10/60)
Diverse range of education	15.0 (9/60)
Missing data	68.3 (41/60)
Context of use of digital technology^c^	
Care or health care	86.7 (52/60)
Social and leisure	11.7 (7/60)
General	3.3 (2/60)
Type of digital technology^c^	
Online platform or app	91.7 (55/60)
Robots or virtual agents	13.3 (8/60)
Smart home sensors or appliance	10 (6/60)
Exergames	5 (3/60)
Wearables	3.3 (2/60)
General software/hardware	1.7 (1/60)

^a^One study in Japan was part of a multisite study with European countries.

^b^For a breakdown of how participants were involved in different design phases and activities, see the full extraction table in multimedia Online Supplementary Material Section 3.

^c^Some digital technologies consisted of more than one component.

Sixty percent of the studies indicated that ethical approval was obtained, whereas 35% did not indicate ethical approval. Of the latter, four studies mentioned that consent was obtained, and three studies did not report obtaining consent even though, in some cases, people with dementia were involved, or video recordings were collected.

Most DTs reported in the studies (86.7%) were designed for the context of care or health care, such as an app to monitor a specific medical condition (e.g., cardiovascular disease) or safety monitoring (e.g., sensors and wearables to detect falls). Only 11.7% of the studies reported the design of DT for social and leisure purposes such as social activities, music entertainment, games, or art.

### Definitions of the Design Process and Involvement of Older Persons

Studies varied in the methodological and conceptual models and frameworks used to define the design process. Most studies referred to user-centered design (43.3%), participatory design (35%), or codesign (13%) as their main conceptual framework. Thirty-six additional conceptual frameworks were used, with most studies (55%) using more than one framework to guide their design process. Older persons were involved in the design process mainly via interviews (61.7%), focus groups (38.3%), design workshops (36.7%), or observations (23.3%). Quantitative questionnaires were often used as a source of additional information (31.7%). An iterative approach in the design process was adopted by 66.7% of the studies and designing with a multidisciplinary approach by 71.7%.

Reviewed studies predominantly (85%) discoursed the importance of involving the end-users throughout the design process, emphasizing that older persons should be involved from the early stages, in an ongoing and iterative manner throughout the whole design process. It was expressed that meaningful involvement throughout the design process may help designers “step outside (their) own experience …, minimize bias, stigmatization and exclusion” (Mertl & Frič, 2019), and “discard assumptions that the people being designed for are similar to the designer” (Di Nuovo et al., 2018). The importance of involving older persons as codesigners was associated by most studies with the utility of designing products which are more fitting into people’s lives and “more likely to result in a feasible and usable end product” (Nguyen et al., 2019).

Furthermore, nearly half of the studies addressed the involvement of older persons in terms of “partnership” and “collaboration,” in which the older persons were recognized as experts of their own experience. This further reflects on how involving older persons relates to issues of power and control related to design processes in general, which is mainly held by the researchers and designers. Involvement was thus described in terms of mitigating power relations and promoting “partnership” through: “transparency” (Span et al., 2018); giving “the end users more control and ownership (in design decisions)” (Willard et al., 2018); and allowing older adults to “have a substantive say in what that outcome is” (Jarke, 2019).

Nevertheless, in 52.9% of the studies which emphasized the importance of involving older persons throughout the whole design process, a contrast was found between these aforementioned “ideological” declarations and the actual practice of involving older persons by the designers and researchers. Older persons were mainly involved in the first phase of empathizing (78.3%) and in the testing and evaluation phase (86.7%). Only 46.7% of studies involved older persons in the actual design and prototyping phase (mainly done by the design team, experts, or other stakeholders). Overall, only 40% of the studies were found to involve older adults throughout the entire design process.

### Forms of Exclusion From the Design Process

Various forms of exclusion practices were identified in 36.7% of the studies in our analysis of sampling methods, inclusion criteria and participant descriptions. Specifically, four studies applied an upper-age limit (e.g., excluding people above 80, with no additional explanation or justification). In five studies, nontech users were excluded, for example, persons with low or no use of touchscreens or computers. Ten studies excluded participants who were not physically active or “healthy enough,” even in cases that the DT was, for example, intended “to support isolated older adults in their homes” (Sidner et al., 2018). Seven studies excluded for psychological reasons (e.g., anxiety or depression) or cognitive decline. Specifically, three out of six studies designing DTs for the use of people with dementia or their caregivers, did not directly involve people with dementia. Usually indicating that “people with different stages of dementia may be confused and unable to communicate their needs and wishes” (Klein et al., 2018). Contrary, some studies emphasized the need for meaningful involvement of people with dementia: “there are still perceptions in society and among professionals that people with dementia are unable and unwilling to reciprocate, and even unworthy of social participation. These perceptions exclude people with dementia from social participation and need to be replaced with more positive ones.” (Span et al., 2018). Notably, several studies were reflective about exclusion of health or cognitive issues as a limitation of their design process.

### Reported Limitations and Identified Biases in the Design Process

Of 31.7% of studies mentioned small sample sizes as a limitation, of which four studies justified small samples as sufficient for the purpose of designing and identifying usability problems. Several additional limitations in the diversity of sampling or identity of the participants were mentioned, such as: not being diverse in terms of culture, ethnicity, lower socioeconomic or education status (six studies); regional or living setting (six studies); not recruiting people with low technological capabilities (four studies); or including only active and healthy participants (two studies).

Noteworthy, the aforementioned limitations were those reported by the studies. Critically reviewing all studies revealed that only 31.7% of studies reported additional characteristics as education (mostly highly educated) or use of DT (25%, which included mainly or only active users of DT, e.g., using touch screens). 80% of the studies reported health characteristics. However, descriptions of health characteristics were often vague, for example, indicating recruitment of “residents with health conditions appropriate for this study―people … affected by general age-related changes, but not suffering from special diseases …” (Duh et al., 2016) or defining functionality in a fuzzy or categorizing manner: “The final system has been tested on both final users (self-sufficient and nonself-sufficient seniors)” (Borelli et al., 2019). In many cases, a clear picture of whether participants were mainly healthy or with diverse health characteristics, could not be determined. More importantly, we found that in 20% of the studies, there seems to be a contrast between the participants who were recruited and the actual intended users. For example, sampling relatively young, active, and healthy persons for a DT intended for people with limited movement and transportation problems or sampling people living independently in the community for a DT intended for people in assisted living communities.

### Discourse About Aging and Implicit and Explicit Manifestations of Ageism

The definition of “old” in the studies, as identified through references on the aging population or inclusion criteria, varied from 50+ (one study), 60+ (16.7%), 65+ (48.3%), 70–75+ (two studies), to 80–85+ (three studies). Only 36.7% of the studies did not define a chronological age to define older persons, and 11.7% used more than one age definition. Specifically, the coding process revealed that 43.3% of the articles used outdated terminology to describe older persons, which are considered categorizing and ageist according to current guidelines (Lundebjerg et al., 2017), such as “elderly,” “elderlies,” “fragility,” “demented,” and “senile.” Moreover, 56.7% of the studies described the process of aging, older persons, and their technological abilities using potentially stereotypical categorizations and generalizations. Main categorizations related to older persons as a group struggling with deteriorated and poor health, low activity levels, cognitive decline, and high dependency on others. Some studies used categorizing descriptions, even while using a definition of 60+ as older age:

“Older adults sometimes forget about whether or not they have completed routine actions and the states of objects that they have interacted with … For many older adults, independent living is possible only with assistance from friends, family, and in-home services that help with their activities of daily living … This is because older adults are likely to have impaired mobility, multiple chronic health conditions, and social and economic limitations.” (Li et al., 2019)

Such mentioning often related to the role of technology to intervene with the so called “problems” of aging, “change” or “make” older adults more active or more independent and reduce costs. Such notions often demonstrated the dominance and power relations of researchers and designers by using patronizing and autonomy-depriving language:

“The technological solutions integrated in (name of DT) have the purpose to assist needy people in the longest stay in their homes in safe conditions, helping them to conduct autonomously most of the activities tied to the satisfaction of their primary needs.” (Borelli et al., 2019)

“Scientific research has been trying to find ICT-enabled solutions to the growing problem of elderly home care … The need to manage several stakeholders and the difficulty of changing some elderly habits can limit the potential of the product service platform.” (Menghi et al., 2019)

Technological abilities were widely portrayed as poor and limited, often without addressing actual facts or literature regarding the abilities of older persons in the specific country (e.g., relating to the prevalence of internet use or digital literacy measurements). In some cases, the technological or communication abilities of older persons were held responsible for unsuccessful outcomes rather than the design process, designers, or final design itself:

“Main insights gained from the studies show, that it is very difficult to keep older people focused on the topics of discussion and that they have often difficulties to clearly present/express their ideas.” (Duh et al., 2016)

“Given the difficulty that older people have in publishing information, contributions supplied directly from older people themselves should not be the major source of such information.” (Gao et al., 2015)

More so, in 21.7% of the studies, design decisions seemed to be influenced by generalizations of older adults as vulnerable, having lower technological capabilities and abilities to participate in a meaningful way in the design process. This often determined the phase and manner in which older adults were involved in the design process:

“In Phase 2, we only tested the prototypes with experts from various disciplines … we feel it is important to not expose a prototype to a potentially vulnerable user group, such as older adults in this case, until it has been fully inspected and walked through by experts.” (Harte et al., 2017)

Notably, 35% of the studies acknowledged older persons as a diverse group of users, whom endure many stereotypes. 15% of the studies explicitly acknowledged ageism as a problem in relation to DT. Namely, that: “significant social stigma exists in this population” (Batsis et al., 2018), and that in contrast with “potential ageist assumptions about the older AT user population and their ICT literacy … studies suggest that older people mostly perceive positively ICT” (Gélinas-Bronsard et al., 2019). Stereotypes about older people were identified as having a potential negative impact on the design process, and this was also related to an implicit motivation that: “companies need to offer products and services that are innovative to set them apart from the competition and to appeal to younger and more tech-savvy users.” (Hurtienne et al., 2015)

Overall, the coding process of the discourse on aging revealed that 33.3% of the included studies were found to contain considerable levels of ageism. Therefore applying stereotypical discourse without balancing or acknowledging the diversity of older users or the complexity of DT use by older persons. Only 38.4% were found to have mixed levels of ageism, meaning that ageist manifestations were identified, along with acknowledgment of the diversity of older persons, reflections on own practice or positive mentioning. Only 28.3% of the studies were found to be with no apparent ageist manifestation and were coded as containing “no ageism.” In these studies, positive aspects of aging, as well as the diversity and heterogeneity of older persons, was emphasized both in relation to needs and wants as well as technological abilities. These articles were more likely to emphasize the importance of involving older adults throughout the design process in terms of the partnership, and made efforts to ensure this. Some papers, coded as “no ageism,” further positively discussed the value of the input received from older persons on their results and reflected on their own surprise from the magnitude, meaningfulness, and importance of older persons’ feedback. Table 2 presents a summary of what we define as manifestations of ageism, errors, and biases in designing DT with older persons, as well as potential best practices identified within the reviewed studies.

**Table 2.** Manifestations of Ageism, Errors, and Biases of Designing DT With Older Persons as Identified in Our Analysis, and Potential Solutions Identified in Reviewed Studies

Design errors and biases		Potential solutions
Exclusion	From the entire design process By upper age inclusion criteria By selection bias toward young, active, healthy, higher educated, “tech-savvy” older persons From specific phases in which feedback from older adults is thought to be unnecessary(e.g., prototyping)	Aspire for an inclusive design process Consider diverse and broad inclusion criteria
User involvement	Only in early or late stages of the design process (usability and evaluation) Single iteration, with no continuity Including only other stakeholders for some stages instead of older persons Low consideration of feedback/No follow up with participants on changes made following feedback Disregarding ethical aspects and obtaining informed consent	Inclusion throughout the whole design process Apply an iterative approach, including older persons View the relationship with older persons as experts and partners Give older people an actual say on the final outcome Sharing control in the decision making Ongoing consent procedures should be applied―especially with people with dementia
Discourse on aging	Use of outdated and stereotyping terms and language Categorizing older persons in terms of frailty, physical and mental decline, and low technological abilities Design decisions based on negative stereotypes and assumptions	Increase awareness, knowledge, and training of the design team and participants on aging and ageist discourse (and guidelines) before the design process Encourage empowerment, view of older persons as experts of their own experience, capable of creativity, and valuable input Engage in self-reflection on ageism Designing in multidisciplinary teams
Speed versus quality	Exclusion or low involvement of older persons due to time and resource considerations and restrains	Consider the benefit of a longer design process comparing to the resources needed to redesign a product with low usability and acceptability Investing time is crucial for meaningful participation that creates inclusion and value Time should also be invested in DT training of participants Funders should require meaningful inclusion and involvement but also calculate the extra time needed for it

*Note*: DT = digital technology.

## Discussion

### Ageism in the Design Process of DT

The goal of this scoping review was to identify the explicit and implicit manifestations of ageism in the design process of DT for older persons. Initially, during the screening process, 19 studies that clearly excluded older persons all together were identified as what can be defined as a form of discrimination. Additional excluded studies reported only on an evaluation of a fully designed DT, with no clear detail regarding how older persons were involved in the design process. However, the inclusion of older persons in the design process, was not found to be a determinant of “no-ageism.” Similar to the findings of Fischer et al. (2020), older persons were found to be involved mainly in the initial phases of empathizing and final phases of usability testing and evaluation, but much less in the actual design phase of ideation, concept development, or prototyping. Additional practices of exclusion were mainly applied in the recruitment and selection of older persons, such as applying upper age inclusion criteria, excluding people with low technological abilities, or not including those who were perceived as not active or healthy enough. Such sampling biases were even more apparent in 20% of the studies, which seemed to have a mismatch between the specific intended users and those who participated in the design process. Forty-five percent of the studies did not provide sufficient detail on the characteristics of the participants. Hence, the sampling and recruitment bias toward younger, active, independent, healthy, tech-savvy, higher-educated, higher socioeconomic status, and ethnically homogenous participants reported by several studies as a limitation, might be wider than found in our analysis.

Stereotypes and prejudice were identified in most studies in the discourse about older persons, their technological (in)abilities, and (in)capacity to participate in the design process. Perhaps the main finding in relation to discourse was the discrepancy found between acknowledging the “ideal” practice of involving older persons throughout the whole design process, as emphasized by almost all studies, and their actual practice of limited involvement. Furthermore, ageism was partly found to influence decisions in the design process.

Unfortunately, ageism is still considered a socially acceptable form of social inequality (Officer & de la Fuente-Núñez, 2018), and though the intentions of researchers and designers are probably good, implicit or subtle manifestations of ageism might interfere with decision-making. Through the lens of CDA, we identified ageism in the discourse about aging. Use of outdated terms such as “elderly” or “demented” could be due to unawareness or differences in daily language, cultural differences, or following common practice. Similar to Vines et al. (2015), older persons were found to be highly categorized in terms of frailty, physical and mental decline, and low technological abilities. Such discourse often persisted even when defining old as 50+ or 60+ and was often found to be paternalistic or autonomy depriving. Fixating on an “interventionist logic” (Peine & Neven, 2019), which defines aging as a problem and DT as a solution, may lead to the perception of what DTs older persons need (close to 90% of designed DTs identified in this review were in the context of care and health care). Similar “medicalization” of needs have also been identified in relation to ableism (Shew, 2020). The process of aging is associated with increased disability, and similarly to our findings, research on “Technoableism” also describes how negative rhetoric on disability in the context of DT may restrain imagination in the design process and *“grounds many technologies being developed to cure or fix disability”* (Shew, 2020, p. 43). While empowerment and good intentions might be in mind, this rhetoric might actually reinforce ableist (or ageist) ideas and expectations about older persons’ capabilities and desires toward using DT.

Consequently, negative images of aging and lower awareness to diversity and stereotypes, may influence the design and decision-making regarding the involvement of older persons (Peine & Neven, 2021). Thus, influencing sampling procedures, level of involvement in iterations, and incorporation of feedback. Noteworthy, our results only describe what we could evidently see reported in the papers. Most probably, the influence of ageism on design decisions are only the “tip of the iceberg.”

It thus seems that merely involving any end user is not *“a simple recipe to increase adoption”* (Gómez & Criado, 2021, p. 85) or to assure an ageist-free or empowering design process. Aspects of stigmatization, exclusion, and dis-empowerment are even more apparent in research and design of DT for people with dementia (Lazar et al., 2017). Reflection is thus needed toward the use of participatory methods, the types of knowledge that are collected through them, and how the role of older persons is viewed in applying these methods.

### From Dominance to Partnership and Shared Control

Decisions made by the design team eventually influence the final “technological artifacts” and how they are used (Peine & Neven, 2021). In making any decision, designers demonstrate their power and control over this process. Through CDA we identified approximately a third of the studies which were defined as nonageist. These studies were found to be more likely to implement participatory principles of sharing or giving up certain amounts of control over decisions in the design process. Furthermore, these studies were more likely to view older persons as innovators and partners and include them iteratively throughout the whole design process. Thus, a link between more positive discourse (or at the very least nonageist discourse) was found with more meaningful participation throughout the design process. Higher levels of participation were often associated with contra to stereotype reports about older persons’ abilities and motivation, higher perceptions of benefit to the design, and satisfaction of the participants.

Sharing control requires a change in the perspective that older persons have different (and valuable) views than other stakeholders (van Boekel et al., 2019) and that they are creative and equal in their ability to become codesigners (Sanders & Stappers, 2008). A difficult task on its own, especially if stereotypical assumptions about the end-users’ abilities to contribute are present. Unfortunately, older persons were evidentially found to be less involved in the actual design process, and when they were, it was many times for a single iteration, with no clear perspective on how their feedback was actually implemented. Most commonly, older persons are positioned as participants rather than partners (Corrado et al., 2020) and are involved only in usability and evaluation phases with advanced prototypes, whereas their influence or say has little impact on the final outcome (Carroll & Rosson, 2007). Notably, there is great variation in the definition of what involvement or participation actually accounts for. A definition that is not well theorized and standardized even with young and healthy users (Vines et al., 2013). We suggest that future definitions and research on the involvement of (older) users take into consideration levels of shared control, the defined relationship between the designers and the users, as well as addressing additional biases detailed and summarized in Table 2.

Interestingly, the aforementioned gap between acknowledging the commercial potential and utility of meaningful involvement of older persons as partners in the design process, is often justified by time and resource constrains (Corrado et al., 2020). Paradoxically, several studies in this review emphasized the importance of investing time in more iterations and higher involvement because the costs of redesigning or making changes to a fully designed product in a later stage might be higher (e.g., Hartzler et al., 2016). Funding and policy makers might as well play an important role in determining inclusion in the design process (Corrado et al., 2020). The majority of studies identified in this review were funded either by public funders or research foundations. Involving end-users is often indicated as a requirement for funding of such projects. However, requiring (and providing training for) meaningful involvement and planning and funding accordingly, might increase the inclusion of older persons in the design process and lead to better design outcomes.

### Limitations

Due to the pervasive nature of ageism, we applied a critical perspective in this study. Nevertheless, we reflect upon and recognize our own potential bias as researchers focusing on ageism and inclusion in design. We conducted this study in a methodological manner and applied an iterative approach in the data analysis following discussions and reflections on our own attitudes. The main limitation, however, is that we could not directly observe the daily practices, interactions, and discourse of researchers and designers with older persons. This implies that more potentially positive aspects were perhaps overlooked. More so, the individual level of the older person as perhaps influenced by self-directed ageism (Köttl et al., 2022; Levy & Banaji, 2002) was also not addressed in this study. Future studies could potentially critically observe the design process and examine the relationship between the discourse on aging of different actors during the design process.

Another limitation pertains to the time frame of this study and the origin of the studies. The findings of this study broaden the findings and search time frame of Fischer et al. (2020). However, the time invested in conducting this study with an extensive discourse analysis may have delayed the publication of these findings from the time of the database search, which ended on January 2020, prior to the coronavirus disease 2019 (Covid-19) pandemic. Ageism was indeed found to be widespread during the pandemic, and issues of equal access and opportunities to use DT were also exacerbated during the Covid-19 pandemic (Chu et al., 2022; Previtali et al., 2020). Nevertheless, this review does not cover potential changes in designing DT for older persons during the pandemic. The specific challenges of designing during the pandemic may as well be an important future inquiry. It may also be that awareness toward ageism has slightly increased in the meantime; however, it is still considered a main societal concern (Officer & de la Fuente-Núñez, 2018), and the implications toward design are still under-recognized. Furthermore, most studies identified in this scoping review were from North America and Europe, as we only included papers in English. Consequently, cultural differences in language, expressions as well as perceptions of values such as respect and equality of older persons were not addressed in this study and could be a line of further inquiry.

## Conclusion

This extensive scoping review identified implicit and explicit manifestations of ageism in the design process of DT. The main manifestation relates to the pervasiveness of how aging is discoursed in general and specifically in the context of DT. Stereotypes and prejudice were found to affect decisions in the design process, and in several cases led to partial or full exclusion from the design process. It was widely agreed by most studies in this review, that involving the end-users in the design process is crucial to ensure designing products that are later on used and meet actual needs and wants. The mismatch identified in this study may imply that in some cases, DTs designed in the reviewed studies did not meet their full potential with the older end-users. Poorly designed DTs might further enhance the negative self-perspectives of older persons in relation to these DTs (and how they are viewed by others). On the positive side, we found evidence that more inclusive design, positive and nonageist discourse, and viewing older persons as partners led to favorable results. Clear requirements and guidance on how to employ language (e.g., by journals or publisher) can foster age-inclusive language in research and policy (Gendron et al., 2016) and consequently increase diversity in the design of DT. This study calls for greater awareness on how ageism influences the design process of DT. Involvement per se does not immediately account for a better design outcome. Designers and researchers involved in designing DT should aspire for meaningful involvement and partnership with older persons.

## Supplementary Material

gnac144_suppl_Supplementary_Material

gnac144_suppl_Supplementary_Tables
