# Supplementary material for: Ageism in the Discourse and Practice of Designing Digital Technology for Older Persons: A Scoping Review
Source: Gerontologist. 2022 Sep 21;63(7):1188–200. doi: 10.1093/geront/gnac144 (PMC10448991; doi:10.1093/geront/gnac144)
Supplement: gnac144_suppl_Supplementary_Material [file gnac144_suppl_supplementary_material.docx]

### Online Supplementary Material

### Supplementary Material Section 1. Key terms and Synonyms

| Design | | Aging | | Digital Technology | |
| --- | --- | --- | --- | --- | --- |
| co-design | TiAb | Frail Elderly | Mesh** | digital* | TiAb |
| codesign | TiAb | Frail | TiAb | tech | TiAb |
| cocreation | TiAb | Aged | Mesh** | technology | TiAb |
| co-creation | TiAb | Aged, 80 and over | Mesh** | technologies | TiAb |
| person-centered AND design | TiAb | aging | Mesh** | information technology | TiAb/ MeSH** |
| person-centered AND research | TiAb | aging | TiAb | ICT | TiAb |
| user-centered AND design | TiAb | ageing | TiAb | "Information communication technology" | TiAb |
| user-centered AND research | TiAb | "older adult" | TiAb | "Information communication technologies" | TiAb |
| "participatory design" | TiAb | "older adults" | TiAb | app | TiAb |
| "participatory research" | TiAb | "older person" | TiAb | apps | TiAb |
| "participatory action design" | TiAb | "older persons" | TiAb | app's | TiAb |
| "participatory action research" | TiAb | "old person" | TiAb | Wearable Electronic Devices | MeSH** |
| community-based AND participatory design | TiAb | "older people" | TiAb | "Wearable Electronic Devices" | TiAb |
| community-based AND participatory research | TiAb | "old people" | TiAb | Wearable* | TiAb |
| community based participatory research | MeSH** | elder* | TiAb | robot* | TiAb |
| Human-centered AND design | TiAb | senior* | TiAb | smartphone | TiAb |
| Human-centered AND research | TiAb | "senior citizen" | TiAb | smartphones | TiAb |
| "design research" | TiAb | "senior citizens" | TiAb | "smart phone" | TiAb |
|  |  | "old age" | TiAb | "smart phones" | TiAb |
|  |  | geriatr* | TiAb | tablet* | TiAb |
|  |  | gerontolog* | TiAb | computer* | TiAb |
|  |  |  |  | smartwatch | TiAb |
|  |  |  |  | smartwatches | TiAb |
|  |  |  |  | "smart watch" | TiAb |
|  |  |  |  | "smart watches" | TiAb |
|  |  |  |  | Gps | TiAb |
|  |  |  |  | mhealth | TiAb |
|  |  |  |  | m-health | TiAb |
|  |  |  |  | ehealth | TiAb |
|  |  |  |  | e-health | TiAb |
|  |  |  |  | gerontech* | TiAb |
|  |  |  |  | geron-tech* | TiAb |
|  |  |  |  | Age-tech* | TiAb |
|  |  |  |  | "smart home" | TiAb |
|  |  |  |  | "smart homes" | TiAb |
|  |  |  |  | smarthome | TiAb |
|  |  |  |  | smarthomes | TiAb |
| *Note*. TiAb = Title and Abstract  ** Mesh terms are only used in PubMed. | | | | | |

### Supplementary Material Section 2. Search string by database and date.

| Data base | Search date | String |
| --- | --- | --- |
| Pubmed | 10/1/2020 | ((((((((((((((((((((cocreation[Title/Abstract]) OR co-creation[Title/Abstract]) OR co-design[Title/Abstract]) OR codesign[Title/Abstract]) OR "design research"[Title/Abstract]) OR ((person-centered[Title/Abstract]) AND design[Title/Abstract])) OR ((person-centered[Title/Abstract]) AND research[Title/Abstract])) OR ((user-centered[Title/Abstract]) AND design[Title/Abstract])) OR ((user-centered[Title/Abstract]) AND research[Title/Abstract])) OR "participatory design"[Title/Abstract]) OR "participatory research"[Title/Abstract]) OR "participatory action design"[Title/Abstract]) OR "participatory action research"[Title/Abstract]) OR ((community-based[Title/Abstract]) AND "participatory design"[Title/Abstract])) OR community-based participatory research[MeSH Terms]) OR ((community-based[Title/Abstract]) AND participatory research[Title/Abstract])) OR ((Human-centered[Title/Abstract]) AND design[Title/Abstract])) OR ((Human-centered[Title/Abstract]) AND research[Title/Abstract]))) AND (((((((((((((((((((((Frail Elderly[MeSH Terms]) OR Frail[Title/Abstract]) OR Aged[MeSH Terms]) OR ((Aged, 80 and over[MeSH Terms]))) OR aging[MeSH Terms]) OR aging[Title/Abstract]) OR ageing[Title/Abstract]) OR "older adult"[Title/Abstract]) OR "older adults"[Title/Abstract]) OR "older person"[Title/Abstract]) OR "older persons"[Title/Abstract]) OR "old person"[Title/Abstract]) OR "older people"[Title/Abstract]) OR "old people"[Title/Abstract]) OR elder*[Title/Abstract]) OR senior*[Title/Abstract]) OR "senior citizen"[Title/Abstract]) OR "senior citizens"[Title/Abstract]) OR "old age"[Title/Abstract]) OR geriatr*[Title/Abstract]) OR gerontolog*[Title/Abstract])) AND (((((((((((((((((((((((((((((((((((((tech[Title/Abstract]) OR technology[Title/Abstract]) OR technologies[Title/Abstract]) OR information technology[MeSH Terms]) OR ICT[Title/Abstract]) OR "Information communication technology"[Title/Abstract]) OR "Information communication technologies"[Title/Abstract]) OR app[Title/Abstract]) OR apps[Title/Abstract]) OR app's[Title/Abstract]) OR Wearable Electronic Devices[MeSH Terms]) OR "Wearable Electronic Devices"[Title/Abstract]) OR Wearable*[Title/Abstract]) OR robot*[Title/Abstract]) OR smartphone[Title/Abstract]) OR smartphones[Title/Abstract]) OR "smart phone"[Title/Abstract]) OR "smart phones"[Title/Abstract]) OR tablet*[Title/Abstract]) OR computer*[Title/Abstract]) OR smartwatch[Title/Abstract]) OR digital[Title/Abstract]) OR "smart watch"[Title/Abstract]) OR smartwatches[Title/Abstract]) OR "smart watches"[Title/Abstract]) OR Gps[Title/Abstract]) OR mhealth[Title/Abstract]) OR m-health[Title/Abstract]) OR ehealth[Title/Abstract]) OR e-health[Title/Abstract]) OR gerontech*[Title/Abstract]) OR geron-tech*[Title/Abstract]) OR Age-tech*[Title/Abstract]) OR "smart home"[Title/Abstract]) OR "smart homes"[Title/Abstract]) OR smarthome[Title/Abstract]) OR smarthomes[Title/Abstract]) |
| Ageline | 17.1.2020 | ((community-based AND "participatory design") OR (community-based AND "participatory research") OR (Human-centered AND design) OR (Human-centered AND research) OR "design research" co-design OR codesign OR cocreation OR co-creation OR (person-centered AND design) OR (person-centered AND research) OR (user-centered AND design) OR (user-centered AND research) OR "participatory design" OR "participatory research" OR "participatory action design" OR "participatory action research") AND (Frail OR aging OR ageing OR "older adult" OR "older adults" OR "older person" OR "older persons" OR "old person" OR "older people" OR "old people" OR elder* OR senior* OR "senior citizen" OR "senior citizens" OR "old age" OR geriatr* OR gerontolog*) AND (digital* OR tech OR technology OR technologies OR information technology OR ICT OR "Information communication technology" OR "Information communication technologies" OR app OR apps OR app's OR "Wearable Electronic Devices" OR Wearable* OR robot* OR smartphone OR smartphones OR "smart phone" OR "smart phones" OR tablet* OR computer* OR smartwatch OR smartwatches OR "smart watch" OR "smart watches" OR Gps OR mhealth OR m-health OR ehealth OR e-health OR gerontech* OR geron-tech* OR Age-tech* OR "smart home" OR "smart homes" OR smarthome OR smarthomes) |
| CINAHL | 13/1/2020 | (TI ( community-based AND "participatory design" ) OR TI ( community-based AND "participatory research" ) OR TI ( Human-centered AND design ) OR TI ( Human-centered AND research ) OR TI "design research" OR TI co-design OR TI codesign OR TI cocreation OR TI co-creation OR TI ( person-centered AND design ) OR TI ( person-centered AND research ) OR TI ( user-centered AND design ) OR TI ( user-centered AND research ) OR TI "participatory design" OR TI "participatory research" OR TI "participatory action design" OR TI "participatory action research" OR AB ( community-based AND "participatory design" ) OR AB ( community-based AND "participatory research" ) OR AB ( Human-centered AND design ) OR AB ( Human-centered AND research ) OR AB "design research" OR AB co-design OR AB codesign OR AB cocreation OR AB co-creation OR AB ( person-centered AND design ) OR AB ( person-centered AND research ) OR AB ( user-centered AND design ) OR AB ( user-centered AND research ) OR AB "participatory design" OR AB "participatory research" OR AB "participatory action design" OR AB "participatory action research") AND (TI Frail OR TI aging OR TI ageing OR TI "older adult" OR TI "older adults" OR TI "older person" OR TI "older persons" OR TI "old person" OR TI "older people" OR TI "old people" OR TI elder* OR TI senior* OR TI "senior citizen" OR TI "senior citizens" OR TI "old age" OR TI geriatr* OR TI gerontolog* OR AB Frail OR AB aging OR AB ageing OR AB "older adult" OR AB "older adults" OR AB "older person" OR AB "older persons" OR AB "old person" OR AB "older people" OR AB "old people" OR AB elder* OR AB senior* OR AB "senior citizen" OR AB "senior citizens" OR AB "old age" OR AB geriatr* OR AB gerontolog*) AND (TI digital* OR TI tech OR TI technology OR TI technologies OR TI information technology OR TI ICT OR TI "Information communication technology" OR TI "Information communication technologies" OR TI app OR TI apps OR TI app's OR TI "Wearable Electronic Devices" OR AB digital* OR AB tech OR AB technology OR AB technologies OR AB information technology OR AB ICT OR AB "Information communication technology" OR AB "Information communication technologies" OR AB app OR AB apps OR AB app's OR AB "Wearable Electronic Devices" OR TI Wearable* OR TI robot* OR TI smartphone OR TI smartphones OR TI "smart phone" OR TI "smart phones" OR TI tablet* OR TI computer* OR TI smartwatch OR TI smartwatches OR TI "smart watch" OR TI "smart watches" OR AB Wearable* OR AB robot* OR AB smartphone OR AB smartphones OR AB "smart phone" OR AB "smart phones" OR AB tablet* OR AB computer* OR AB smartwatch OR AB smartwatches OR AB "smart watch" OR AB "smart watches" OR TI Gps OR TI mhealth OR TI m-health OR TI ehealth OR TI e-health OR TI gerontech* OR TI geron-tech* OR TI Age-tech* OR TI "smart home" OR TI "smart homes" OR TI smarthome OR TI smarthomes OR AB Gps OR AB mhealth OR AB m-health OR AB ehealth OR AB e-health OR AB gerontech* OR AB geron-tech* OR AB Age-tech* OR AB "smart home" OR AB "smart homes" OR AB smarthome OR AB smarthomes) |
| ACM Digital | 16/1/2020 | [[[Publication Title: community-based] AND [Publication Title: "participatory design"]] OR [[Publication Title: community-based] AND [Publication Title: "participatory research"]] OR [[Publication Title: human-centered] AND [Publication Title: design]] OR [[Publication Title: human-centered] AND [Publication Title: research]] OR [Publication Title: "design research"] OR [Publication Title: co-design] OR [Publication Title: codesign] OR [Publication Title: cocreation] OR [Publication Title: co-creation] OR [[Publication Title: person-centered] AND [Publication Title: design]] OR [[Publication Title: person-centered] AND [Publication Title: research]] OR [[Publication Title: user-centered] AND [Publication Title: design]] OR [[Publication Title: user-centered] AND [Publication Title: research]] OR [Publication Title: "participatory design"] OR [Publication Title: "participatory research"] OR [Publication Title: "participatory action design"] OR [Publication Title: "participatory action research"]] AND [[Publication Title: frail] OR [Publication Title: aging] OR [Publication Title: ageing] OR [Publication Title: "older adult"] OR [Publication Title: "older adults"] OR [Publication Title: "older person"] OR [Publication Title: "older persons"] OR [Publication Title: "old person"] OR [Publication Title: "older people"] OR [Publication Title: "old people"] OR [Publication Title: elder*] OR [Publication Title: senior*] OR [Publication Title: "senior citizen"] OR [Publication Title: "senior citizens"] OR [Publication Title: "old age"] OR [Publication Title: geriatr*] OR [Publication Title: gerontolog*]] AND [[Publication Title: digital*] OR [Publication Title: tech] OR [Publication Title: technology] OR [Publication Title: technologies] OR [Publication Title: information technology] OR [Publication Title: ict] OR [Publication Title: "information communication technology"] OR [Publication Title: "information communication technologies"] OR [Publication Title: app] OR [Publication Title: apps] OR [Publication Title: app's] OR [Publication Title: "wearable electronic devices"] OR [Publication Title: wearable*] OR [Publication Title: robot*] OR [Publication Title: smartphone] OR [Publication Title: smartphones] OR [Publication Title: "smart phone"] OR [Publication Title: "smart phones"] OR [Publication Title: tablet*] OR [Publication Title: computer*] OR [Publication Title: smartwatch] OR [Publication Title: smartwatches] OR [Publication Title: "smart watch"] OR [Publication Title: "smart watches"] OR [Publication Title: gps] OR [Publication Title: mhealth] OR [Publication Title: m-health] OR [Publication Title: ehealth] OR [Publication Title: e-health] OR [Publication Title: gerontech*] OR [Publication Title: geron-tech*] OR [Publication Title: age-tech*] OR [Publication Title: "smart home"] OR [Publication Title: "smart homes"] OR [Publication Title: smarthome] OR [Publication Title: smarthomes]] AND [[[Abstract: community-based] AND [Abstract: "participatory design"]] OR [[Abstract: community-based] AND [Abstract: "participatory research"]] OR [[Abstract: human-centered] AND [Abstract: design]] OR [[Abstract: human-centered] AND [Abstract: research]] OR [Abstract: "design research"] OR [Abstract: co-design] OR [Abstract: codesign] OR [Abstract: cocreation] OR [Abstract: co-creation] OR [[Abstract: person-centered] AND [Abstract: design]] OR [[Abstract: person-centered] AND [Abstract: research]] OR [[Abstract: user-centered] AND [Abstract: design]] OR [[Abstract: user-centered] AND [Abstract: research]] OR [Abstract: "participatory design"] OR [Abstract: "participatory research"] OR [Abstract: "participatory action design"] OR [Abstract: "participatory action research"]] AND [[Abstract: frail] OR [Abstract: aging] OR [Abstract: ageing] OR [Abstract: "older adult"] OR [Abstract: "older adults"] OR [Abstract: "older person"] OR [Abstract: "older persons"] OR [Abstract: "old person"] OR [Abstract: "older people"] OR [Abstract: "old people"] OR [Abstract: elder*] OR [Abstract: senior*] OR [Abstract: "senior citizen"] OR [Abstract: "senior citizens"] OR [Abstract: "old age"] OR [Abstract: geriatr*] OR [Abstract: gerontolog*]] AND [[Abstract: digital*] OR [Abstract: tech] OR [Abstract: technology] OR [Abstract: technologies] OR [Abstract: information technology] OR [Abstract: ict] OR [Abstract: "information communication technology"] OR [Abstract: "information communication technologies"] OR [Abstract: app] OR [Abstract: apps] OR [Abstract: app's] OR [Abstract: "wearable electronic devices"] OR [Abstract: wearable*] OR [Abstract: robot*] OR [Abstract: smartphone] OR [Abstract: smartphones] OR [Abstract: "smart phone"] OR [Abstract: "smart phones"] OR [Abstract: tablet*] OR [Abstract: computer*] OR [Abstract: smartwatch] OR [Abstract: smartwatches] OR [Abstract: "smart watch"] OR [Abstract: "smart watches"] OR [Abstract: gps] OR [Abstract: mhealth] OR [Abstract: m-health] OR [Abstract: ehealth] OR [Abstract: e-health] OR [Abstract: gerontech*] OR [Abstract: geron-tech*] OR [Abstract: age-tech*] OR [Abstract: "smart home"] OR [Abstract: "smart homes"] OR [Abstract: smarthome] OR [Abstract: smarthomes]] |
| Web of science core collection | 13/1/2020 | ((TOPIC: ((((((((((((((((community-based AND "participatory design") OR (community-based AND "participatory research")) OR (Human-centered AND design)) OR (Human-centered AND research)) OR "design research" co-design) OR codesign) OR cocreation) OR co-creation) OR (person-centered AND design)) OR (person-centered AND research)) OR (user-centered AND design)) OR (user-centered AND research)) OR "participatory design") OR "participatory research") OR "participatory action design") OR "participatory action research") AND TOPIC: ((((((((((((((((Frail OR aging) OR ageing) OR "older adult") OR "older adults") OR "older person") OR "older persons") OR "old person") OR "older people") OR "old people") OR elder*) OR senior*) OR "senior citizen") OR "senior citizens") OR "old age") OR geriatr*) OR gerontolog*)) AND TOPIC: (((((((((((((((((((((((((((((((((((digital* OR tech) OR technology) OR technologies) OR information technology) OR ICT) OR "Information communication technology") OR "Information communication technologies") OR app) OR apps) OR app's) OR "Wearable Electronic Devices") OR Wearable*) OR robot*) OR smartphone) OR smartphones) OR "smart phone") OR "smart phones") OR tablet*) OR computer*) OR smartwatch) OR smartwatches) OR "smart watch") OR "smart watches") OR Gps) OR mhealth) OR m-health) OR ehealth) OR e-health) OR gerontech*) OR geron-tech*) OR Age-tech*) OR "smart home") OR "smart homes") OR smartphone) OR smarthome)) |
| Psycinfo | 14/01/2020 | (TI ( (community-based AND "participatory design") OR (community-based AND "participatory research") OR (Human-centered AND design) OR (Human-centered AND research) OR "design research" co-design OR codesign OR cocreation OR co-creation OR (person-centered AND design) OR (person-centered AND research) OR (user-centered AND design) OR (user-centered AND research) OR "participatory design" OR "participatory research" OR "participatory action design" OR "participatory action research" ) OR AB ( (community-based AND "participatory design") OR (community-based AND "participatory research") OR (Human-centered AND design) OR (Human-centered AND research) OR "design research" co-design OR codesign OR cocreation OR co-creation OR (person-centered AND design) OR (person-centered AND research) OR (user-centered AND design) OR (user-centered AND research) OR "participatory design" OR "participatory research" OR "participatory action design" OR "participatory action research" )) AND (TI ( Frail OR aging OR ageing OR "older adult" OR "older adults" OR "older person" OR "older persons" OR "old person" OR "older people" OR "old people" OR elder* OR senior* OR "senior citizen" OR "senior citizens" OR "old age" OR geriatr* OR gerontolog* ) OR AB ( Frail OR aging OR ageing OR "older adult" OR "older adults" OR "older person" OR "older persons" OR "old person" OR "older people" OR "old people" OR elder* OR senior* OR "senior citizen" OR "senior citizens" OR "old age" OR geriatr* OR gerontolog* )) AND (TI ( digital* OR tech OR technology OR technologies OR information technology OR ICT OR "Information communication technology" OR "Information communication technologies" OR app OR apps OR app's OR "Wearable Electronic Devices" OR Wearable* OR robot* OR smartphone OR smartphones OR "smart phone" OR "smart phones" OR tablet* OR computer* OR smartwatch OR smartwatches OR "smart watch" OR "smart watches" OR Gps OR mhealth OR m-health OR ehealth OR e-health OR gerontech* OR geron-tech* OR Age-tech* OR "smart home" OR "smart homes" OR smarthome OR smarthomes ) OR AB ( digital* OR tech OR technology OR technologies OR information technology OR ICT OR "Information communication technology" OR "Information communication technologies" OR app OR apps OR app's OR "Wearable Electronic Devices" OR Wearable* OR robot* OR smartphone OR smartphones OR "smart phone" OR "smart phones" OR tablet* OR computer* OR smartwatch OR smartwatches OR "smart watch" OR "smart watches" OR Gps OR mhealth OR m-health OR ehealth OR e-health OR gerontech* OR geron-tech* OR Age-tech* OR "smart home" OR "smart homes" OR smarthome OR smarthomes )) |
| Co-design journal | 16/01/2020 | ((community-based AND "participatory design") OR (community-based AND "participatory research") OR (Human-centered AND design) OR (Human-centered AND research) OR "design research" co-design OR codesign OR cocreation OR co-creation OR (person-centered AND design) OR (person-centered AND research) OR (user-centered AND design) OR (user-centered AND research) OR "participatory design" OR "participatory research" OR "participatory action design" OR "participatory action research") AND (Frail OR aging OR ageing OR "older adult" OR "older adults" OR "older person" OR "older persons" OR "old person" OR "older people" OR "old people" OR elder* OR senior* OR "senior citizen" OR "senior citizens" OR "old age" OR geriatr* OR gerontolog*) AND (digital* OR tech OR technology OR technologies OR information technology OR ICT OR "Information communication technology" OR "Information communication technologies" OR app OR apps OR app's OR "Wearable Electronic Devices" OR Wearable* OR robot* OR smartphone OR smartphones OR "smart phone" OR "smart phones" OR tablet* OR computer* OR smartwatch OR smartwatches OR "smart watch" OR "smart watches" OR Gps OR mhealth OR m-health OR ehealth OR e-health OR gerontech* OR geron-tech* OR Age-tech* OR "smart home" OR "smart homes" OR smarthome OR smarthomes) |

### Supplementary Material Section 3. Full list of extracted variables, quoted segments, and coding (available as a separate Excel spreadsheet file).
